# Study of heteroaggregation and properties of sol-gel AlOOH–Fe_3_O_4_ composites

**DOI:** 10.1016/j.heliyon.2020.e05825

**Published:** 2020-12-28

**Authors:** Vasily I. Mikhaylov, Ilia S. Martakov, Evgeny Yu. Gerasimov, Petr A. Sitnikov

**Affiliations:** a Institute of Chemistry, Federal Research Centre, Komi Science Centre of the Ural Branch of the Russian Academy of Sciences, 48 Pervomyaskaya Street, 167982 Syktyvkar, Russia; b Boreskov Institute of Catalysis SB RAS, Pr. Lavrentieva 5, Novosibirsk, 630090, Russia

**Keywords:** Magnetite, Boehmite, Composites, Chromium, Heteroaggregation

## Abstract

In this work, AlOOH–Fe_3_O_4_ powder nanocomposites for Cr(VI) adsorption were obtained for the first time using oppositely charged boehmite and citric acid modified magnetite sols. The process of heteroaggregation of oppositely charged AlOOH and Fe_3_O_4_ nanoparticles was also studied as one of the stages in the preparation of adsorption active material. Сomposition, surface area, porous structure, thermal and surface properties, adsorption efficiency, and regenerability of nanocomposites were investigated using a wide range of analytical methods. It is noted that a low content of magnetite (2 wt.%) in the AlOOH–Fe_3_O_4_ composite promotes an increase in the surface area, weakly affects the Cr(VI) adsorption capacity, and imparts magnetic properties to the composite. Low cost, simplicity of preparation, high Cr(VI) adsorption capacity (up to 21 mg/g), and stability in cyclic use are the advantages of the obtained nanocomposites in comparison with similar systems. They can easily be separated from the purified liquid using a permanent magnet due to their magnetic properties.

## 1 Introduction

Magnetic materials, including those based on magnetite Fe_3_O_4_, have a very wide range of applications today. So, magnetite is used in spintronics and electronics, engineering, catalysis, chemical and mining industries (production of lubricants, systems for recording and storing information, magnetic fluid separators) due to its magnetic properties [1, 2, 3]. Biocompatibility of magnetite allows using it in biomedicine to enhance contrast and increase the diagnostic sensitivity of MRI, targeted delivery of therapeutic agents to living objects, and hyperthermia using an alternating magnetic field [4].

Nevertheless, colloidal solutions stable in physiological media and highly efficient adsorbents based on unmodified magnetite are difficult to obtain due to the near-neutral value of the isoelectric point (IEP), high density, and low concentration of surface functional groups. There are several ways to avoid these disadvantages: the surface of magnetite particles is coated with substances of various nature (organic surfactants, oxides with a porous structure, etc.) [5, 6], or composite powders containing the second component with an improved texture are synthesized [7].

The Fe_3_O_4_/SiO_2_ system is one of the most studied composite systems [6, 8]. Similar structures have shown themselves well to immobilize proteins [9], as biosensors [8], drug carriers [10], and adsorbents [6]. At the same time, little attention is paid in the scientific literature to the study of the physicochemical and functional properties of the boehmite (γ-AlOOH) – magnetite (Fe_3_O_4_) system. This system is no less interesting since γ-AlOOH exhibits a high specific surface area, good chemical stability, biocompatibility, and low cost [11]. It also exhibits antioxidant activity [12]. The composite can be promising as a magnetically separable adsorbent or catalyst due to the magnetic characteristics of magnetite.

Published works describing the magnetite-boehmite system are devoted chiefly to the core-shell structure. So, in Ref. [11] mesoporous γ-AlOOH@Fe_3_O_4_ magnetic nanomicrospheres were synthesized. Based on studies of texture (178.9 m^2^/g) and magnetic (46.6 emu/g) characteristics, the authors proposed the use of this system in catalysis and adsorption separation. The prospects of the Pd/Al–AlOOH@Fe_3_O_4_ system for catalytic reduction of rhodamine B, 4-nitrophenol, methyl orange, and for the Heck coupling reaction were shown in Ref. [13, 14].

A study of the boehmite-magnetite composite system obtained by the sol-gel method was published recently in Ref. [7]. The authors performed mixing of the same (positively) charged sols, as a result the system was aggregately stable over the entire range of ratios, and the final products provided a specific surface area of up to 391 m^2^/g. At the same time, the surface properties of nanoparticles (surface charge and aggregative stability to electrolytes or nanoparticles of a different nature), can be radically changed by altering the functional composition of the surface. The opposite charge on the surface of the oxides promotes the process of heteroaggregation, as well as the formation of a stronger bond between the components. The interactions between heterogeneous colloids often encountered in practice are more difficult to study and understand. They are interesting both from a fundamental point of view (to understanding the mechanisms of interaction) and a practical one (deposition and removal of toxic nanoparticles). Heterocoagulation processes are rarely studied as a stage in the synthesis of functional materials.

The development of new effective adsorbents for heavy metals is one of the most relevant areas of research due to the increased attention to environmental problems. Despite the high interest in this topic, many fundamental questions remain regarding the relationship between the sorption capacity and the method of producing adsorbents, their composition and porosity, the nature of adsorbate and surface functional groups, and also concerning the mechanisms of adsorption.

Hexavalent chromium compounds (anionic forms CrO_4_^2-^, HCrO_4_^-^, and Cr_2_O_7_^2-^ depending on concentration and pH) are one of the most dangerous substances for organisms at low concentrations. They have a carcinogenic effect, can penetrate the cell membrane and react with intracellular material, and also cause respiratory diseases, skin allergies, hemolysis, cancer and lung fibrosis [15, 16, 17, 18]. According to guidelines from the World Health Organization, the maximum allowable limit for chromium in drinking water is 50 μg/L [18]. Sources of chromium in natural water are enterprises of leather, textile, woodworking, galvanic industries, and metallurgy.

Ref. [7] is one of the few studies of the Cr(VI) adsorption capacity in the boehmite-magnetite system. The authors have shown that this composite has a high adsorption capacity, reaching 62.4 mg/g. However, this value was achieved in a strongly acidic environment (at pH 2). It is known that magnetite and boehmite can be dissolved under these conditions.

In this work, AlOOH–Fe_3_O_4_ powder composites for adsorption of highly toxic Cr(VI) compounds from aqueous media were obtained using oppositely charged boehmite and citric acid modified magnetite sols. Important characteristics of the systems planned to be used as adsorbents (composition, surface area, porous structure, adsorption efficiency, and regenerability) were studied. The process of heteroaggregation of oppositely charged boehmite and magnetite nanoparticles was also studied as one of the stages in the preparation of adsorption active material.

## 2 Experimental

**Figure 1:**
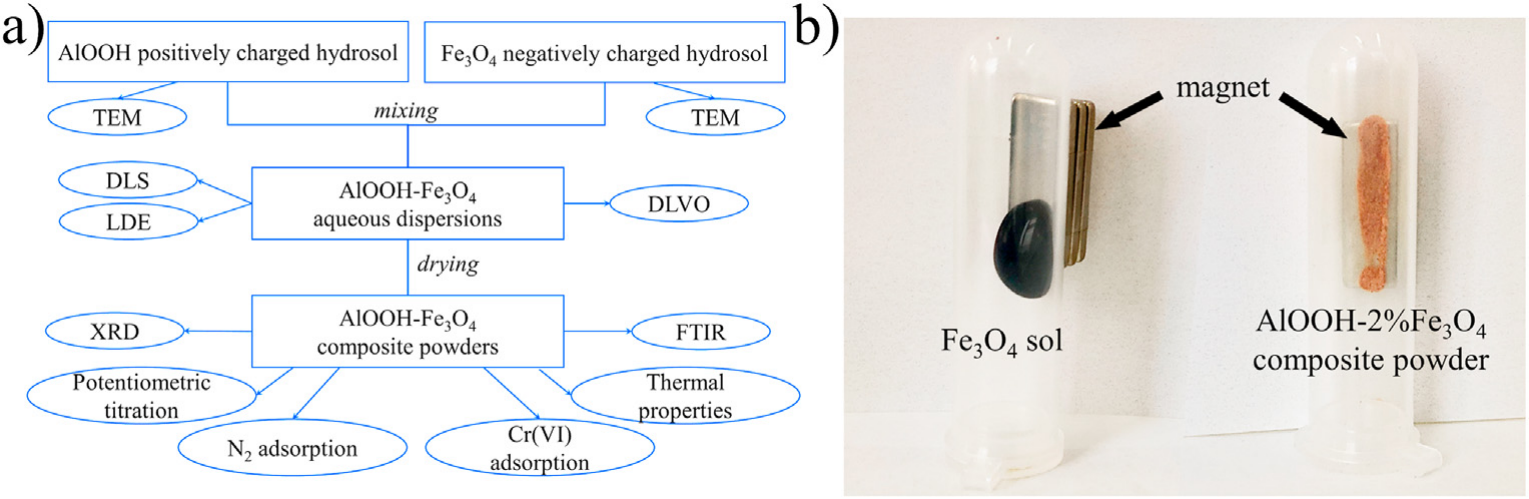
The scheme of preparation and studying of AlOOH–Fe_3_O_4_ dispersions and powders (a). Photo of magnetite hydrosol and composite powder in the presence of a magnet (b).

The scheme of the experiments and the methods used is presented in Figure 1a.

### 2.1 Sample preparation

The synthesis of boehmite sol was carried out according to the known method with minor changes [19]. Aluminum isopropoxide (3.3 g) (Acros Organics, >98 %) hydrolysis was performed by adding it to 100 ml of water preheated to 85 °C under vigorous stirring for 15 min giving suspension of white precipitate. At the next step, the dispersion was subjected to ultrasonic treatment (Sapphire UZV-5.7 ultrasonic bath, 37 kHz, 150 W) for 4 h. During the ultrasonic treatment, the sol was heated to the temperature of 75 °C at the end of treatment. As a result, stable alumina sol was obtained.

The synthesis of citrate-stabilized magnetite sol was carried out according to a modified method proposed by us earlier in Ref. [20]. As a result, we have obtained Fe_3_O_4_ magnetite hydrosol, where particles cannot be magnetically separated from an aqueous medium (Figure 1b).

The mass fraction of the solid phase in the sols was determined by gravimetric analysis. The sols were mixed in various calculated volumetric ratios, followed by drying (at 105 °C) them to obtain AlOOH–Fe_3_O_4_ composite powders.

### 2.2 Heteroaggregation experiments

To study the process of heteroaggregation, a calculated amount of magnetite sol was added to a fixed amount of AlOOH in an aqueous dispersion. Mixing of boehmite and magnetite sols was carried out in various volume ratios; the mass fraction of magnetite (relative to the mass of the solid phase) was varied in the range of 0–100 wt.%. The changes of the zeta potentials and particle size were determined by dynamic light scattering (DLS) and laser Doppler electrophoresis (LDE) (Malvern ZetaSizer Nano ZS, light scattering angle of 173°, 4 mW He/Ne laser, 633 nm) at 25 °C in a DTS1070 disposable capillary cell (Malvern). All measurements were carried out after 1 min of intensive mixing of the components and were repeated at least three times.

The studies were carried out both without a background electrolyte and in the presence of KCl (1 mM).

The energy of pair interaction of Fe_3_O_4_–Fe_3_O_4_, AlOOH–Fe_3_O_4_, and AlOOH–AlOOH was determined by the DLVO (Derjaguin–Landau–Verwey–Overbeek) theory, which takes into account such components of the pair interaction energy (*U*) of particles as the energy of electrostatic repulsion (*U*_e_) and the molecular attraction energy (*U*_m_):

(1) *U* = *U*_e_ + *U*_m_.

The energy of molecular attraction was calculated according to the Hogg-Healy-Fuerstenau formula [21, 22]:

(2) $$U_{e}=\frac{\pi \varepsilon \varepsilon_{0}r_{1}r_{2}(\phi_{1}^{2}+\phi_{2}^{2})}{r_{1}+r_{2}}\left\{\frac{2\phi_{1}\phi_{2}}{\phi_{1}^{2}+\phi_{2}^{2}}ln\left[\frac{1+exp\{-\kappa h\}}{1-exp\{-\kappa h\}}\right]+ln\left[1+exp\{-2\kappa h\}\right]\right\},$$

where *ε* is the permittivity of the dispersive medium; *ε*_*0*_ is the permittivity of vacuum, F/m; *r*_*i*_ are particle radii, m; *ϕ*_*i*_ are the zeta potentials of the particles, V; *κ* is the Debye parameter, m^−1^; and *h* is the distance between the surfaces of the particles, m. Since lamellar boehmite nanoparticles are quite polydisperse in shape and size, the calculations were carried out on the assumption of their spherical shape using the hydrodynamic radius determined by the DLS method. Moreover, due to rotations of the particles in the disperse medium, their shape in solution is close to spherical, what also assumed in the DLS calculations from raw light scattering data.

The energy of molecular attraction of the particles was calculated by the equation [23]:

(3) $$U_{m}=-\frac{A}{6}\left\{\frac{2r_{1}r_{2}}{h^{2}+2r_{1}h+2r_{2}h}+\frac{2r_{1}r_{2}}{h^{2}+2r_{1}h+2r_{2}h+4r_{1}r_{2}}+ln\;\frac{h^{2}+2r_{1}h+2r_{2}h}{h^{2}+2r_{1}h+2r_{2}h+4r_{1}r_{2}}\;\right\},$$

where *A* is the Hamaker constant. While no value of Hamaker constant was reported for CA-Fe_3_O_4_, the *A* value of 2 · 10^−20^ J was used [24, 25]. This value is in the range typical for other similar surfactants on magnetite, including oleic (2.0 · 10^−20^ J) and humic acids (1.38–4.0 · 10^−20^ J). The value of the Hamaker constant was taken as 12.6 · 10^−20^ J for AlOOH particles [26].

To calculate the energy of pair interaction of different nature particles, the *A*_132_ value was determined by the equation:

(4) $$A_{132}=\left(\sqrt{A_{11}}-\sqrt{A_{33}}\right)\cdot \left(\sqrt{A_{22}}-\sqrt{A_{33}}\right),$$

where *A*_11_ and *A*_22_ are the Hamaker constants for interacting AlOOH and Fe_3_O_4_ particles, respectively [27], and *A*_33_ is the Hamaker constant for water [28].

### 2.3 Characterization

The magnetite and boehmite nanoparticles were studied using a JEM-2010 (JEOL, Japan) transmission electron microscope (TEM). Fourier transform infrared (FTIR) spectra of all samples were collected on a Prestige 21 (Shimadzu, Japan) spectrometer. X-ray diffraction (XRD) patterns were obtained using an XRD-6000 (Shimadzu, Japan) diffractometer with Cu *Kα* radiation. The nitrogen adsorption/desorption measurements of the adsorbents were performed at 77 K using Nova 1200e system (Quantachrome). Thermal analysis was performed on STA 409 PC/PD (NETZSCH) instrument with a heating rate of 20 °C/min in the temperature range 25–1200 °C.

Acid-base surface properties of powders were studied using an ATP-02 (Aquilon) automatic potentiometric titrator. The titration was conducted in argon atmosphere in polypropylene vessels with constant concentrations of background electrolyte (1 mM KCl). A detailed description of the experiment is described in Ref. [12].

### 2.4 Adsorption experiments

Stock solutions with a Cr(VI) concentration of 10, 20, and 30 mg/L were obtained by dissolving a calculated quantity of potassium dichromate (K_2_Cr_2_O_7_, 99.5%, Panreac) in water. The isotherms of adsorption of Cr(VI) ions on the AlOOH–Fe_3_O_4_ composite powders were investigated by dispersing a 10 mg sample in 10 ml of a Cr(VI) solution. The resulting suspensions were stirred for 1 h. The pH values of the initial Cr(VI) solutions varied in the range of 3.0–5.0. After experiments, the dispersions were filtered through 0.2 μm pore size membrane filters. Chromium concentration was determined by the 1,5-diphenylcarbazide method [29].

## 3 Results and discussions

### 3.1 Properties of boehmite and magnetite sols

**Figure 2:**
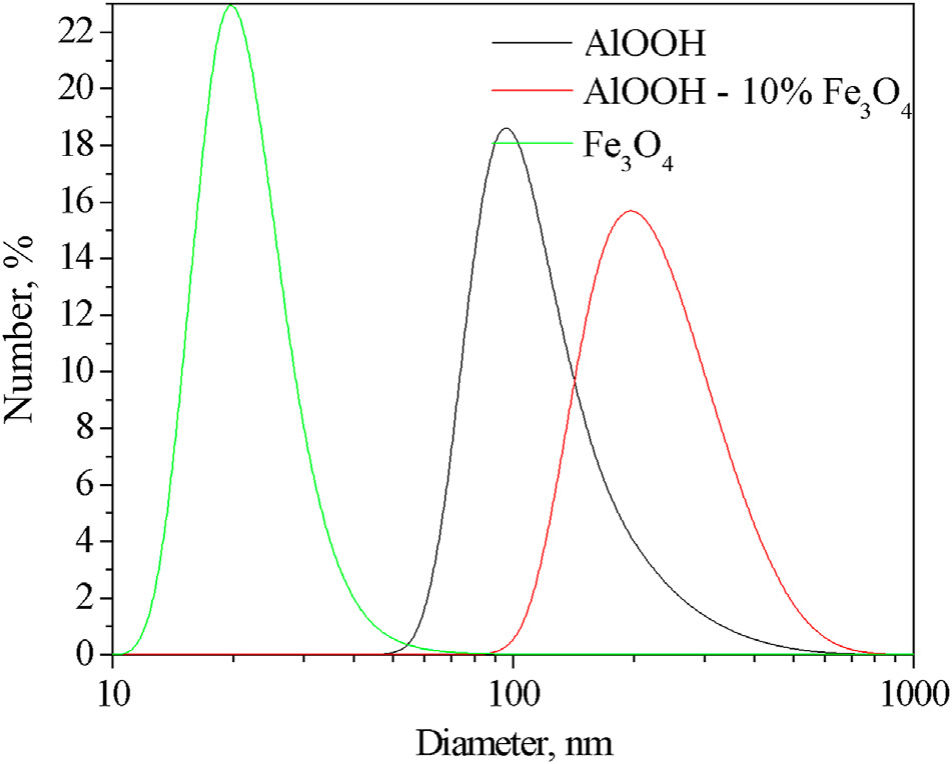
Particle size distributions of the AlOOH, Fe_3_O_4_, and AlOOH–10%Fe_3_O_4_ sols.

**Figure 3:**
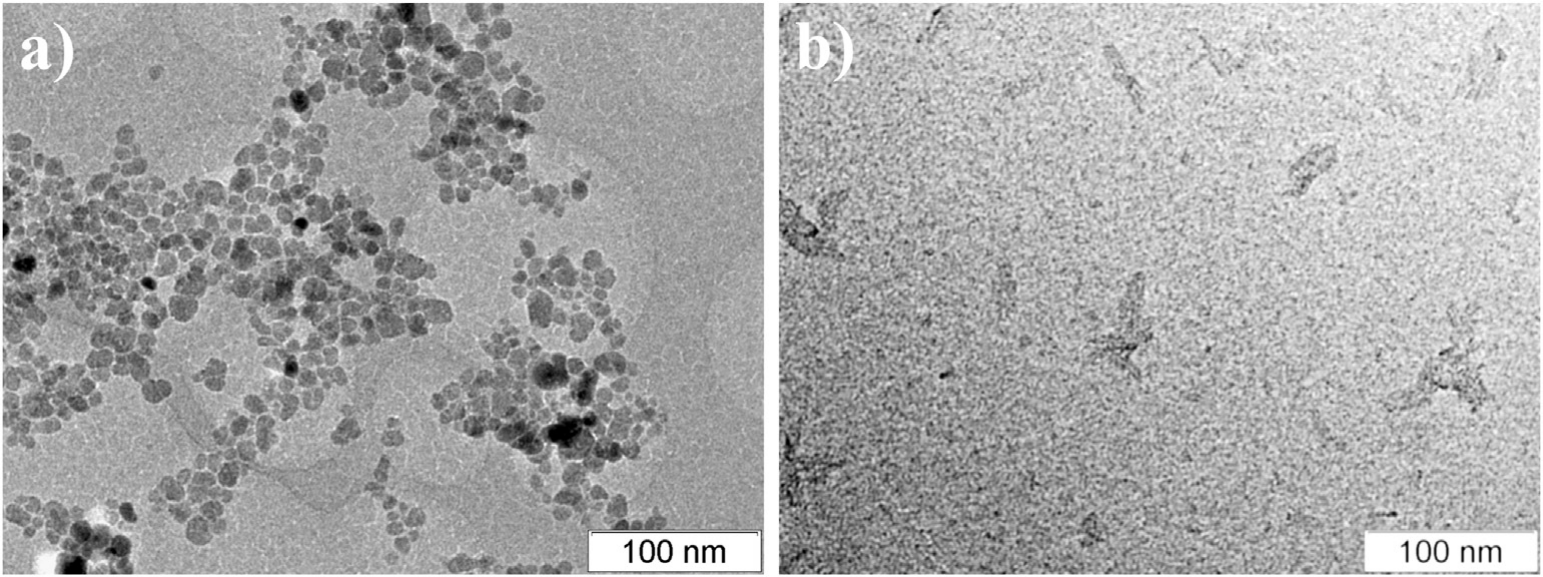
TEM images of the Fe_3_O_4_ (a) and AlOOH (b) nanoparticles.

The main characteristics of the obtained sols are given in Table 1. It was noted that both sols are characterized by a monomodal particle size distribution (Figure 2). Nanoparticles in both sols are characterized by higher Z-average hydrodynamic and number mean diameters (Figure 2, Table 1) than the sizes of solid nanoparticles determined by the direct TEM method (Figure 3, Table 1). Magnetite nanoparticles have a spherical shape with an average size of about 9 nm (Figure 3a), while boehmite nanoparticles have a lamellar shape (Figure 3b). The sizes of Fe_3_O_4_ nanoparticles correspond to the characteristic range of sizes of nanoparticles obtained by coprecipitation method (8–12 nm, Table 2).

**Table 1:** Parameters of as-synthesized hydrosols of boehmite and magnetite.

Parameter	γ-AlOOH	Fe_3_O_4_
Z-Average hydrodynamic diameter, nm	214 ± 3	57.4 ± 0.9
Peak size (by intensity), nm	239 ± 89	76 ± 46
Number mean, nm	146 ± 24	21.8 ± 2.2
Peak size (by number), nm	129 ± 66	20 ± 6
Zeta potential, mV	+43.1 ± 1.0	-26.7 ± 0.8
Average diameter (TEM), nm	-	9 ± 3
pH_IEP_	9.7	2.30
pH_PZC_	7.23	3.84
Mass fraction, %	0.61	1.48

**Table 2:** Comparison of particle sizes of magnetite obtained by coprecipitation.

Preparation method	Average diameter (TEM), nm	Crystalline size (XRD), nm	Hydrodynamic diameter (DLS), nm	Zeta potential, mV	Reference
US-assisted coprecipitation, CA-coated a	9	12.6	21.8	-26.7	This work
US-assisted coprecipitation	10	10	33	32	[30]
coprecipitation	11.92	10.3			[31]
coprecipitation	11.22	11.00			[32]
coprecipitation (Khalil's procedure)	10.93			-12.87	[33]
coprecipitation	8–15	17.65	100		[34]
coprecipitation, CA-stabilized	7–10		162.4		[35]

a US: ultrasound; CA: citric acid.

On the one hand, the nanoparticles in the dispersion medium are in an agglomerated state even after ultrasonic treatment (especially boehmite). On the other hand, the DLS method also takes into account the solvation shell around the nanoparticles.

**Figure 4:**
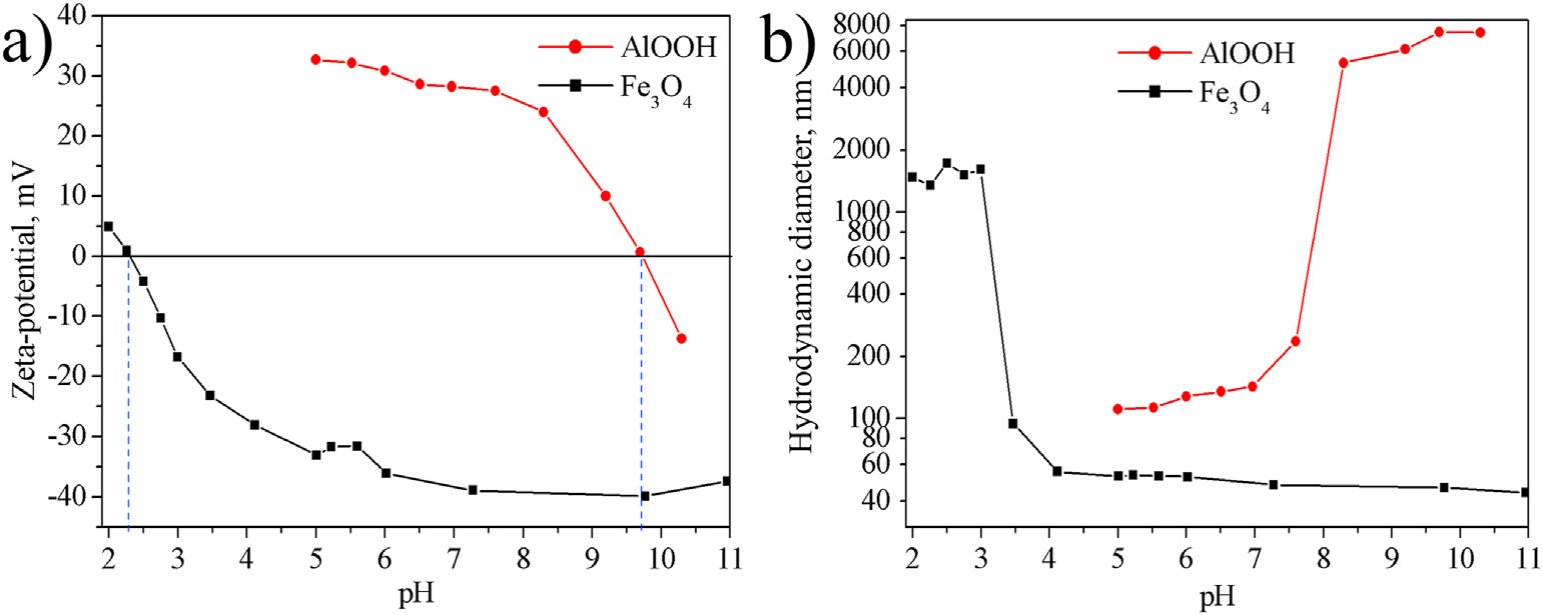
The pH dependences of the zeta potential (a) and particle size (b) of the Fe_3_O_4_ and AlOOH.

The zeta potentials of both sols at circumneutral pH exhibit different signs since the values of the isoelectric points of the sols are markedly different. The pH_IEP_ of boehmite sol is in the alkaline range, while in magnetite it is in the acidic range (Table 1, Figure 4a). The positive surface charge of boehmite is formed due to the protonation of surface hydroxyl groups:

(5) -OH_(surf)_ + H^+^ ↔ –OH_2_^+^_(surf)_.

A negative charge on the surface of citrate-stabilized magnetite is formed due to the ionization of surface carboxyl groups that are not associated with the surface of magnetite [20]:

(6) -COOH_(surf)_ + OH^−^ ↔ -COO^-^_(surf)_ + H_2_O.

As the pH of the sol approaches the pH_IEP_, the zeta potential tends to zero (Figure 4a), and the hydrodynamic particle size increases (Figure 4b). Magnetite sol with pH ≤ 3 (zeta potential > -20 mV) and boehmite one with pH > 8 (zeta potential <25 mV) are aggregatively unstable. The hydrodynamic diameter is greater than 1 μm in these pH ranges, as a result of which the dispersions rapidly coagulate.

Thus, sols are oppositely charged in a wide range of pH 2.3–9.7, which contributes to their heteroaggregation.

### 3.2 Heteroaggregation of nanoparticles in the aqueous media

**Figure 5:**
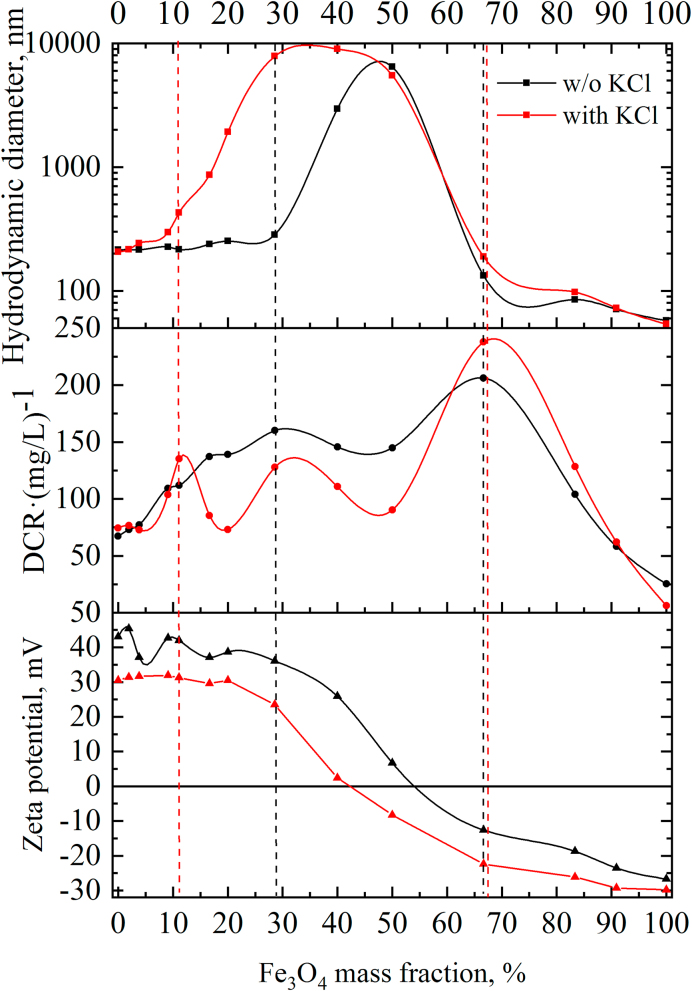
Dependence of the particle size, derived count rate, and zeta potential on Fe_3_O_4_ content in AlOOH–Fe_3_O_4_ system.

**Figure 6:**
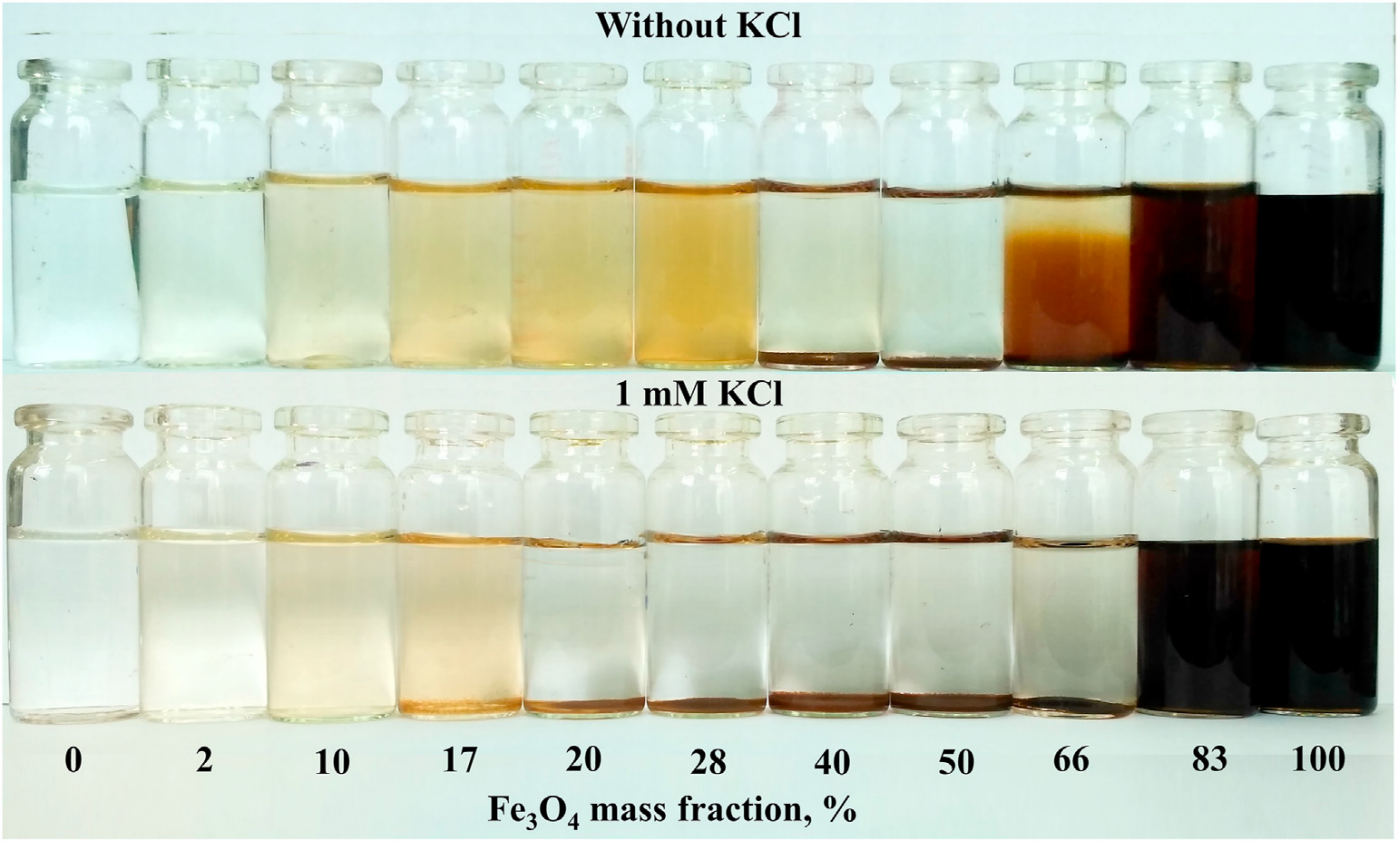
Photo of AlOOH–Fe_3_O_4_ dispersions in the absence (above) and the presence (below) of KCl.

When sols are mixed, the negatively charged Fe_3_O_4_ nanoparticles attach to the positively charged AlOOH nanoparticles via electrostatic attraction. As the concentration of Fe_3_O_4_ increases, the zeta potential decreased from ~ +43 mV to -27 mV, passing through zero at a magnetite concentration of ~55 wt.% (Figure 5). High stability of the zeta potential and hydrodynamic diameter values up to 30 wt.% Fe_3_O_4_ was noted. The sizes of aggregates began to increase significantly starting from 40 wt.% Fe_3_O_4_. Dispersed systems with a mass fraction of magnetite of up to 30% and more than 80% are sedimentation-stable, while sedimentation is observed in the range from 40 to 70 wt.% Fe_3_O_4_ (Figure 6).

Derived count rate (DCR) is a convenient parameter for evaluating heteroaggregation, although it is practically not used in the scientific literature for these purposes. DCR is a parameter calculated in the Zetasizer Nano software, which is the scattering intensity that can be measured in the absence of a laser attenuation filter [36]. DCR is a useful measure for comparing signal strength from different samples: a higher DCR usually indicates larger particles or higher concentration, or both. Since the mass fraction of boehmite is constant, the total mass fraction of the solid phase in the solution increases with the addition of a magnetite sol. In this regard, the DCR divided by the total concentration of the solid phase was evaluated (DCR·(mg/L)^−1^). In the absence of electrolyte, an increase in DCR is observed in the range of 0–30 wt.% Fe_3_O_4_, coupled with an increase in particle size. Further, DCR is slightly reduced in the range of 30–50 wt.% Fe_3_O_4_, although a strong increase in particle size is evident. This can be attributed to the settling of large agglomerates, causing a decrease in the concentration of particles in the solution (Figure 6). A further increase in DCR in the range up to 70 wt.% Fe_3_O_4_ can be attributed to the preservation of large agglomerates in the solution volume that are resistant to precipitation due to electrostatic repulsion. A gradual decrease in DCR is observed when the mass fraction of magnetite is more than 70%, associated with a decrease in the average particle size.

The mass fraction of magnetite nanoparticles at which the zeta potential passes through zero decreased to 42 wt.% in the presence of 1 mM background electrolyte. Also, the ranges of stability of hybrid systems are narrowing. Coagulation occurs in the range of 17–70 wt.% Fe_3_O_4_; dispersions with a magnetite content of up to 10 and more than 80 wt.% are stable. The presence of an electrolyte leads to a partial decrease in the surface charge of nanoparticles, which contributes to the formation of larger heteroaggregates (Figure 5). The change in DCR also confirms a wider range of instability, since a sharp decrease in DCR associated with coagulation is observed starting from 17 wt.% Fe_3_O_4_ (Figure 5).

**Figure 7:**
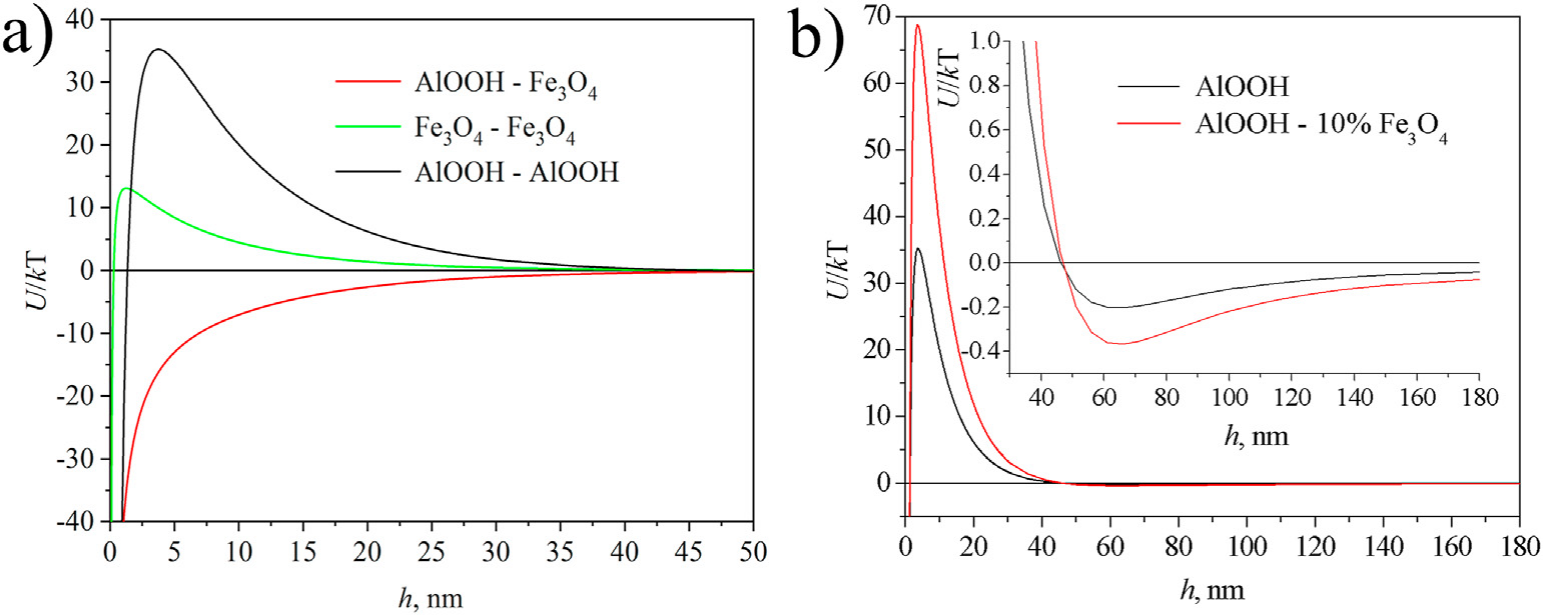
Pairwise interaction curves of Fe_3_O_4_–Fe_3_O_4_, AlOOH–AlOOH, and Fe_3_O_4_–AlOOH (a). Pairwise interaction curves of AlOOH nanoparticles and AlOOH–10%Fe_3_O_4_ hybrid particles (b). The ionic strength is taken equal to 0.001 M.

Theoretical calculations of the interaction energy of particles were carried out according to the DLVO theory. There is no potential barrier on the curves of pairwise interaction of nanoparticles of different nature (Fe_3_O_4_–AlOOH) (Figure 7). This indicates the rapid adhesion of particles of various nature to each other due to electrostatic forces. A potential barrier preventing the approach of nanoparticles and coagulation is present in the pairwise interaction curves of particles of the same nature (Fe_3_O_4_–Fe_3_O_4_, AlOOH–AlOOH). This is confirmed by the high aggregative stability of the as-synthesized sols. The value of the zeta potential practically did not change upon the addition of a small number of magnetite nanoparticles (10 wt.%) to the boehmite sol, but a slight increase in the size of agglomerates was observed (Figure 5). Electrostatic repulsive forces also prevail over attractive forces during the interaction of such larger aggregates (Figure 7b). An almost twofold increase in the height of the potential barrier with a simultaneous increase in the depth of the secondary potential minimum is observed on the pairwise interaction curves of composite particles as compared to the initial boehmite. It is known that the presence of a secondary potential well with a sufficiently high energy repulsion barrier is favorable for the formation of gel-like structures [37]. It is obvious that this process is facilitated by an increase in particle concentration during the drying process.

Thus, primary heteroaggregates are formed at low Fe_3_O_4_ concentrations. These heteroaggregates still retain the high positive zeta potential, so no coagulation occurs. As the Fe_3_O_4_ concentration increases, the value of the zeta potential and the electrostatic repulsion energy of heteroaggregates decrease, and further aggregation occurs. As a result, coagulation is observed in systems with a zeta potential close to zero. The second range of stability is observed when the concentration of magnetite exceeds the critical concentration of heterocoagulation. Here, stability is determined by the excess of negatively charged magnetite nanoparticles in comparison with positively charged boehmite particles.

### 3.3 Characterization of solids

**Figure 8:**
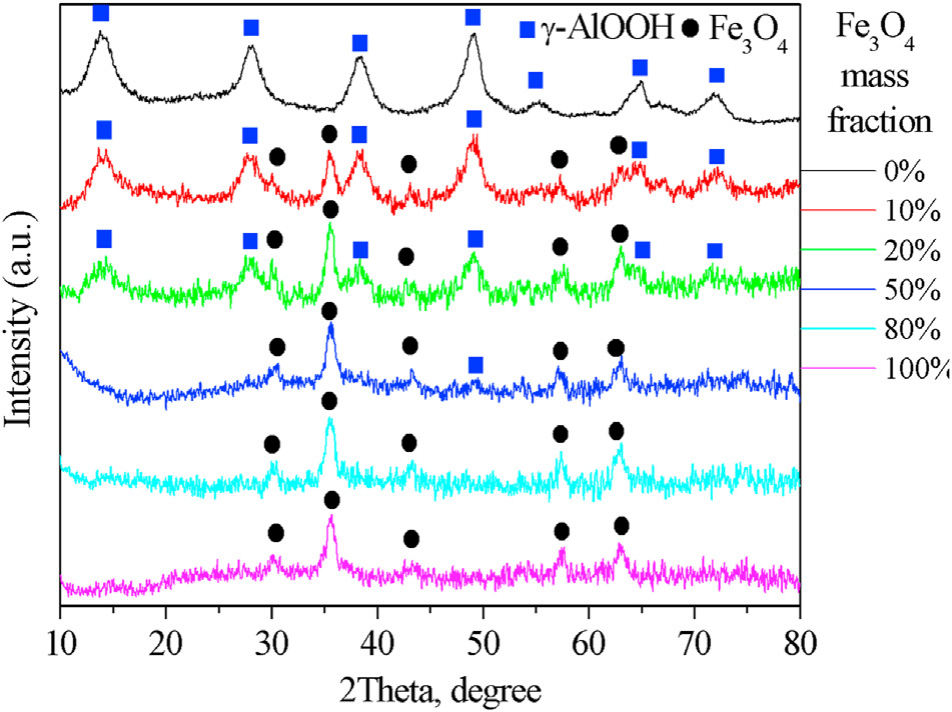
X-ray diffraction patterns of AlOOH–Fe_3_O_4_ composite powders with different mass fractions of magnetite.

It is noted that all magnetite-containing powders exhibit magnetic properties and are attracted to the permanent magnet (Figure 1b). According to the results of XRD analysis (Figure 8), only boehmite (γ-AlOOH, JCPDS No. 21-1307) and magnetite (Fe_3_O_4_, JCPDS No. 19-0629) phases are present in the powders. The average crystallite size is 5.0 nm for the boehmite and 12.6 nm for the magnetite according to the calculation by the Debye-Scherrer formula (*D* = 0.90*λ*/*β*cos*θ,* where *λ* is X-ray wavelength, *β* is the line broadening measured at half-height, and *θ* is the Bragg angle of the particles) [38].

**Figure 9:**
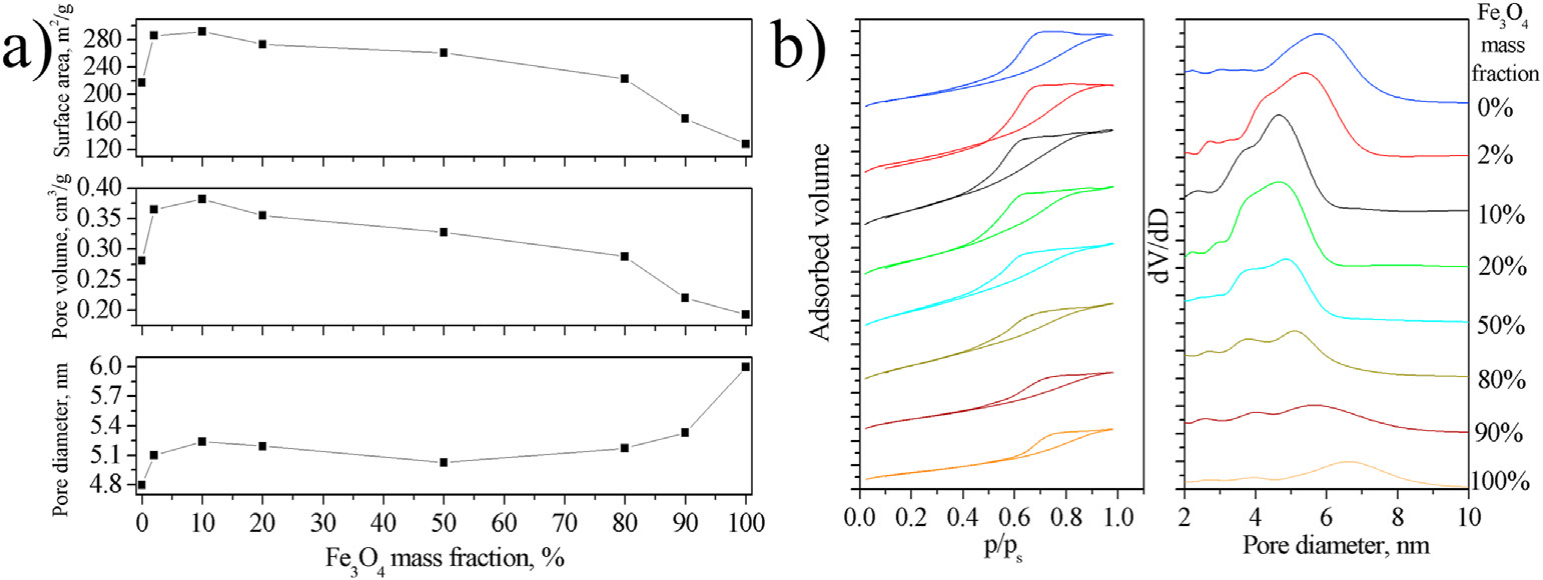
(a) Change in surface area, average pore volume, and average pore diameter depending on the composition of AlOOH–Fe_3_O_4_ composite powders. (b) Nitrogen sorption isotherms and pore size distribution curves in AlOOH–Fe_3_O_4_ composite powders with different mass fractions of magnetite.

It was indicated that AlOOH–Fe_3_O_4_ powders provide a high specific surface area (129–292 m^2^/g). A sharp increase in the surface area (up to 292 m^2^/g) is observed with an increase in the mass fraction of magnetite up to 10 wt.%. A further increase in the mass fraction of magnetite leads to a gradual decrease in the surface area (down to 129 m^2^/g for pure Fe_3_O_4_) (Figure 9a). The increase in surface area is associated with an increase in volume and average pore diameter.

The presence of a small number of magnetite nanoparticles apparently causes a decrease in the degree of agglomeration during drying and an increase in the dispersity of powders. The particle sizes were estimated based on the data of physical adsorption of nitrogen in the approximation of the spherical shape of particles by the formula [39]:

(7) *d* = 6/*S·ρ*,

where *d* – the average diameter of particle (m), *S* – surface area (m^2^/g), *ρ* – material density (g/m^3^).

According to the calculation, the average diameter of particles is 9.2 and 9.0 nm for pure boehmite and magnetite, respectively. The diameter of magnetite particles coincides with the TEM results because of the close to the spherical shape of the nanoparticles. The average particle size of boehmite is 7.0 nm if the change in density of the system in the presence of 2 wt.% magnetite is neglected. This value approaches the crystallite size values of boehmite according to XRD analysis.

Adsorption-desorption isotherms (Figure 9b) of all powders are of type IV(a) (according to the IUPAC classification), which are typical for mesoporous materials. Isotherms have an H2-type hysteresis loop associated with capillary condensation of nitrogen in mesopores with a complex structure. The maximum in the pore size distribution curve (Figure 9b) gradually decreases from 6.1 to 4.5 nm with an increase in the magnetite content to 20 wt.%, but then increases to 6.8 nm.

Apparently, the addition of a small number of magnetite nanoparticles (up to 10 wt.%) leads to the formation of stable structures such as Fe_3_O_4_@AlOOH. Magnetite nanoparticles attached to the boehmite surface do not allow AlOOH particles to adhere strongly to each other during drying. As a result, powders with a highly porous structure and increased surface area are formed.

**Figure 10:**
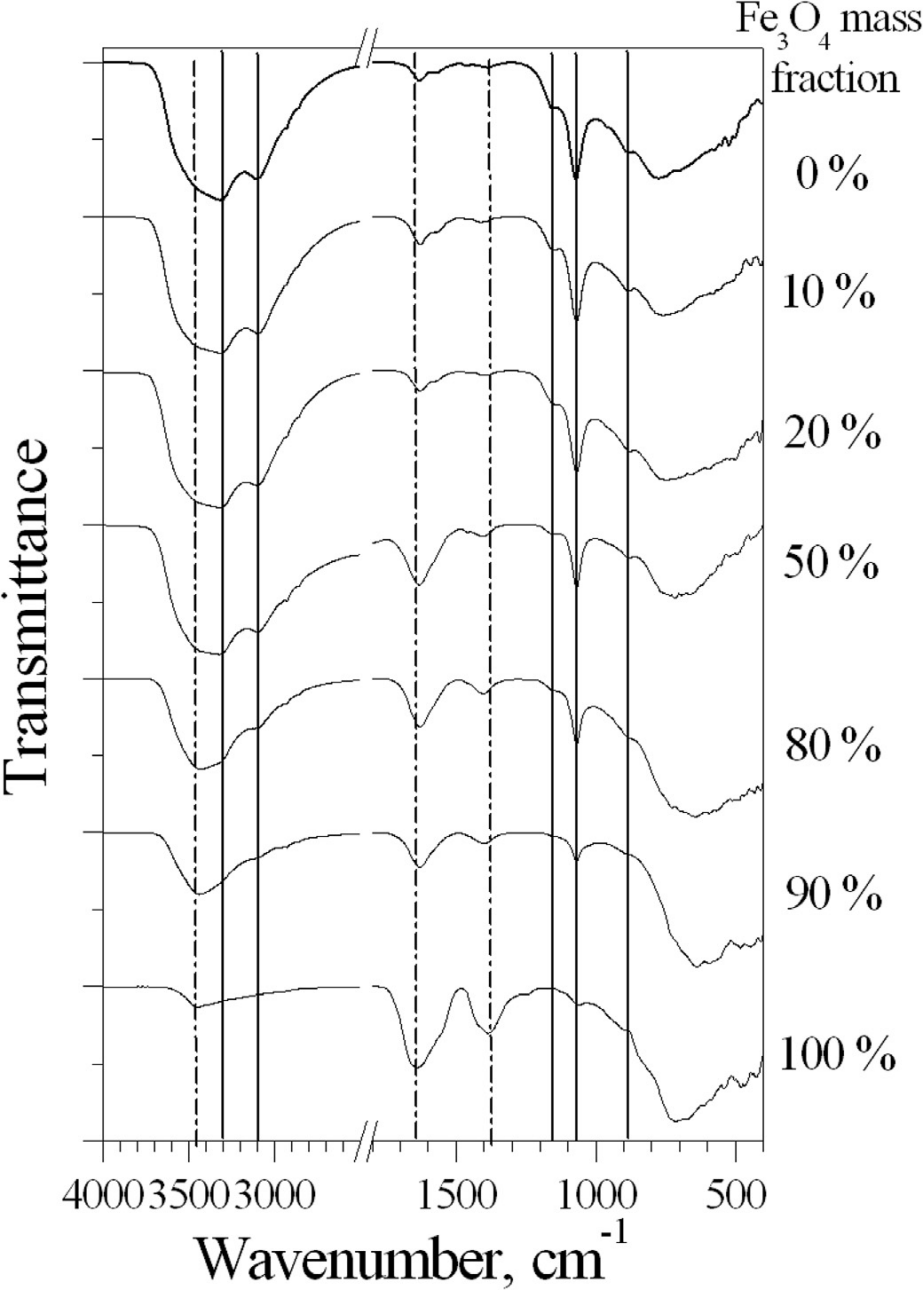
FTIR spectra of boehmite, magnetite, and AlOOH–Fe_3_O_4_ composite powders. The solid vertical line corresponds to bands characteristic for AlOOH, the dashed line corresponds to bands characteristic for magnetite.

The FTIR spectra of Fe_3_O_4_ (Figure 10) have two absorption bands at 450 and 700 cm^−1^, which indicates the formation of magnetite structure [40]. The absorption band at 3443 cm^−1^ is due to O–H stretching mode of vibration of surface hydroxyl groups or adsorbed water on the magnetite surface. The intense band appears at 1385 cm^−1^ (symmetric stretching vibration of C=O), and very strong band 1638 cm^−1^ (asymmetric stretching mode of COO^−^ unit). Thus, in the magnetite, the carboxyl (-COOH) groups of citric acid strongly coordinate to iron cations on Fe_3_O_4_ surface to form the coating. Uncoordinated carboxylate groups of citric acid extended into the water medium are responsible for the formation of a negative charge in the case of the Fe_3_O_4_ sol.

FTIR spectra of AlOOH show peaks of the asymmetric and symmetric valence stretches of the interlayer OH groups at 3316 and 3100 cm^−1^. The band at 1628 cm^−1^ are attributed to H–O–H bands. The band at 1070 cm^−1^ corresponds to the δs Al–O–H mode of boehmite and the bands at 400–900 cm^−1^ are attributed to the Al–O bands of boehmite [41].

It was shown that the FTIR spectra of composites containing 10 and 20 wt.% magnetite are almost identical to the spectrum of the initial boehmite, that is, magnetite bands practically do not appear in the spectrum of these composites. This is consistent with a very weak change in the zeta potential up to 30 wt.% Fe_3_O_4_ (Figure 4a). At the magnetite content is more than 50 wt.%, the intensity of bands in the range 2500–3800 cm^−1^ and the band at 1070 cm^−1^ decrease due to a decrease in the total number of –OH groups. Also, when the magnetite content is more than 50 wt%, the bands of symmetric and asymmetric stretching vibration of C=O and COO^−^ unit characteristic of magnetite appear on the spectra of the composites. The characteristic bands for boehmite are retained on the spectra up to its 10 wt% content in the composite. It was noted that the addition of 10 wt.% boehmite leads to a sharp decrease in the intensity of the bands of symmetric and asymmetric stretching vibration of C=O and COO^−^ unit. The imposition of bands of 3316 cm^−1^ for boehmite and 3443 cm^−1^ for magnetite is observed with an increase in the magnetite content. This affects the shape of the broad band of vibration of surface hydroxyl groups. In general, no significant band shifts were detected in the FTIR spectra of the composites, which could indicate the interaction of components through citrate groups.

**Figure 11:**
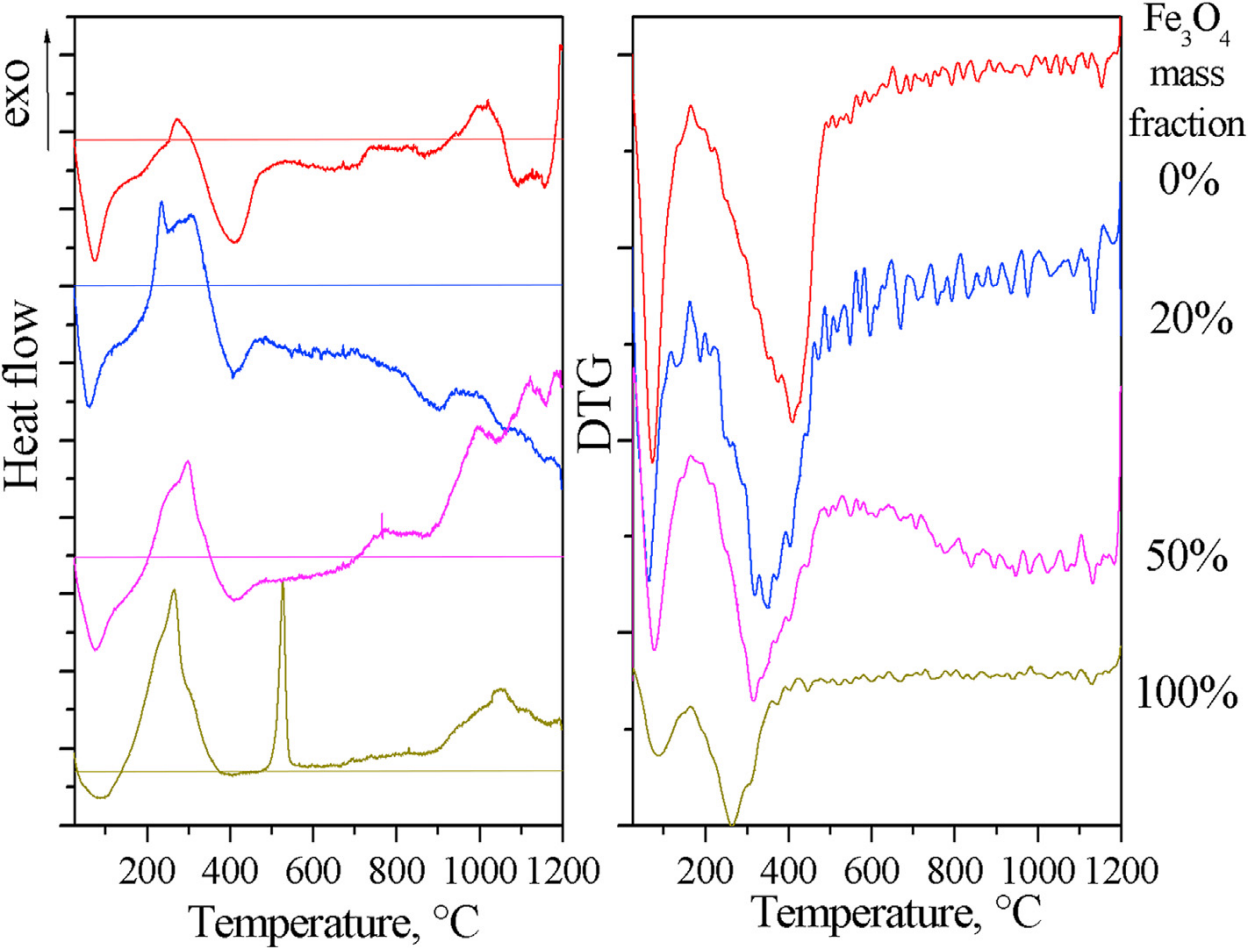
Heat flow and DTG curves of AlOOH–Fe_3_O_4_ composite powders with different mass fractions of magnetite.

The thermal properties of the composites were studied by synchronous thermal analysis. The heat flow curves (Figure 11) of all samples exhibit an endothermic effect (with a minimum of about 70 °C), accompanied by mass loss and associated with the removal of adsorbed water. The endothermic effect of aluminum oxyhydroxide dehydration (2AlOOH → Al_2_O_3_ + H_2_O) with a minimum of about 410 °C, also accompanied by a decrease in mass, is observed for boehmite-containing samples. The intensity of this effect naturally decreases with a decrease in the boehmite fraction in the samples. The exothermic effect at about 260 °C, most likely associated with the thermal-oxidative degradation of citrates, is observed in pure magnetite powder. This effect is also present in all magnetite-containing samples, but its intensity decreases with a decrease in the mass fraction of magnetite. The intense exothermic effect at 530 °C for pure magnetite is not accompanied by a change in mass and is most likely associated with crystallization of hematite α-Fe_2_O_3_ [40].

Potentiometric titration is one of the most accessible and widely used methods for studying acid-base equilibria on the surface of oxides in water-salt systems. There are several mathematical models for calculating the parameters of a double electric layer, in particular, «pK spectroscopy» [42]. The main provisions of the model are described by us earlier in [20]. This approach allows not only to calculate the surface complexation constants pK_i_, but also to determine the concentration of acid-base centers, per 1 g of the sample (q_i_, mmol/g).

**Figure 12:**
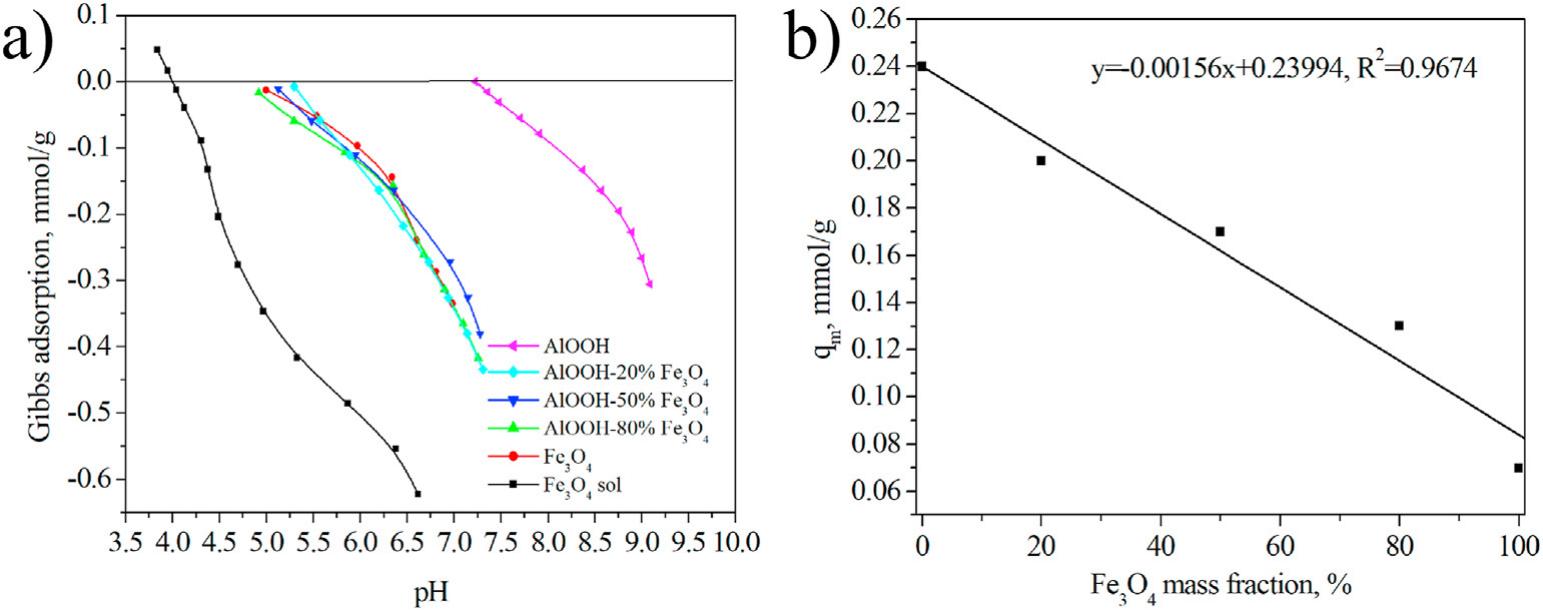
Change in net specific surface proton excess on the surface of AlOOH and magnetite-containing samples depending on the pH in 0.001 M KCl (a). Change in the number of the acid-base center q_M_ for AlOOH–Fe_3_O_4_ composite powders depending on Fe_3_O_4_ mass fraction (b).

Figure 12a shows the change in net specific surface proton excess on adsorption of ions from pH for boehmite and various magnetite-containing systems. Based on these dependencies, the constants of surface complexation (pK_i_) and the number of acid-base centers corresponding to these equilibria (q_i_) were calculated (Table 3).

**Table 3:** The values of pK_i_ and q_i_ for AlOOH and magnetite-containing samples.

Sample	pK_M_, Δ±0.05	q_M_, Δ±0.01, mmol/g	pK_2_, Δ±0.05	q_2_, Δ±0.01; mmol/g
AlOOH	7.10	0.24	8.60	0.32
AlOOH-20% Fe_3_O_4_	5.45	0.20	7.10	0.41
AlOOH-50% Fe_3_O_4_	5.30	0.17	7.10	0.41
AlOOH-80% Fe_3_O_4_	4.70	0.13	6.95	0.51
Fe_3_O_4_	4.70	0.07	6.80	0.51
Fe_3_O_4_ sol	3.95	0.81	6.50	0.05

It is noted that the curves of changes in net specific surface proton excess from pH for xerosols differ markedly from the curves for sols (Figure 12a). The coincidence of the pK_i_ values (3.95) for the Fe_3_O_4_ sol with citric acid ionization constants given in Ref. [20] suggests that the stabilization of magnetite nanoparticles with citric acid occurs as a result of its orientation in the Stern plane of the double electric layer. The acid molecules interact with the base centers of magnetite with the formation of covalent bonds during the drying of the sol. This leads to the formation of new acid-base centers with pK_M_ = 4.7 (responsible for the process $-\text{S}-\text{OH}+{\text{M}}^{+}↔-\text{S}-{\text{O}}^{-}\cdots {\text{M}}^{+}+{\text{H}}^{+}$) and pK_2_ = 6.8 (responsible for the deprotonation of the surface OH group: $-\text{S}-\text{OH}↔-\text{S}-{\text{O}}^{-}+{\text{H}}^{+}$). The pK_2_ value for Fe_3_O_4_ is close to that for the initial sol.

It was noted that the position of the titration curves of powdered magnetite and all magnetite-containing composites is almost identical, but it differs from the position of the titration curve for pure boehmite. A stepwise change in the constants of surface complexation is observed, depending on the ratio of the components. The surface complexation constants of composites with 20–50 wt.% Fe_3_O_4_ have an intermediate value between the constants for magnetite and boehmite. For AlOOH–80%Fe_3_O_4_ sample, pK_i_ value is close to its value for magnetite. It was noted that the dependence of the number of acid-base sites (q_M_) on the magnetite content is linear (Figure 12b).

The results obtained suggest a relatively large particle of boehmite is the core of composite particles for all the considered ratios of components. The adhesion of smaller spherical magnetite nanoparticles occurs on the surface of the boehmite particle due to acid-base (donor-acceptor) interactions.

### 3.4 Adsorption behavior of solids toward Cr(VI)

**Figure 13:**
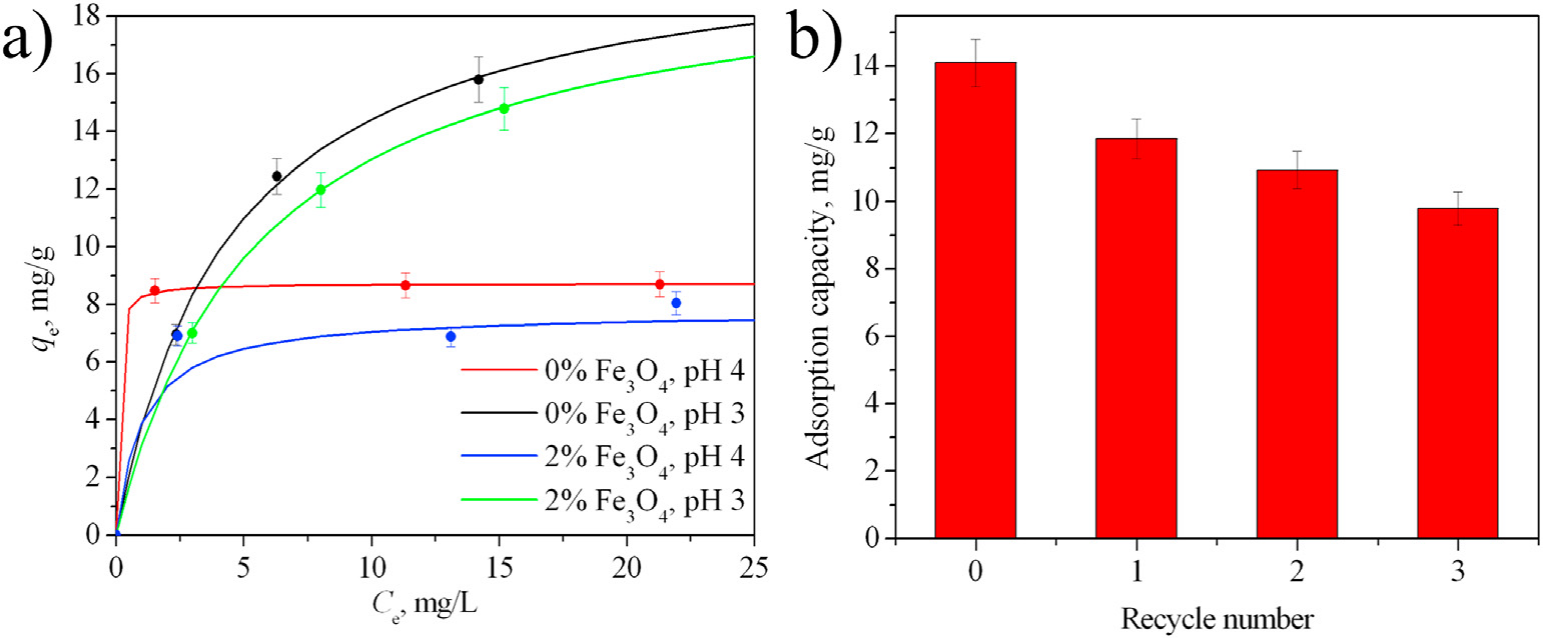
(a) Adsorption isotherms for Cr(VI) on AlOOH–Fe_3_O_4_ composite powders with different mass fractions of magnetite at different pH. The lines correspond to the Langmuir model. (b) Change in the Cr(VI) adsorption on an AlOOH–2%Fe_3_O_4_ sample upon cyclic use (pH 3.0, C_0_(Cr^VI^) = 30 mg/L, desorption using a 0.01 M NaOH solution).

The Cr(VI) adsorption properties of powdered composites were investigated. The effect of the composition of the powders and the pH of the medium on the adsorption capacity was studied. The adsorption capacity was determined from the results of processing the adsorption isotherms using the Langmuir model (Figure 13a, Table 4). At pH 5.0, the sorption capacity reaches 4.9 mg/g for pure boehmite and decreases sharply to 0.5 mg/g in the presence of 2 wt.% magnetite. With a decrease in pH to 4.0, the sorption capacity is 8.7 mg/g for pure AlOOH, 7.8 mg/g for AlOOH–2%Fe_3_O_4_, and 5.6 mg/g for AlOOH–10%Fe_3_O_4_. A significant decrease in the adsorption capacity with a small addition of magnetite is consistent with the conclusion that the boehmite surface is covered with magnetite nanoparticles.

**Table 4:** Parameters and correlation coefficients of the Langmuir and Freundlich equations for some samples.

Adsorbent	pH	Langmuir			Freundlich		
		Adsorption capacity *q*_m_, mg/g	*K*_*L*_, (dm^3^/mg)	R^2^	*K*_*F*_	1/*n*	R^2^
AlOOH	3.0	21.0	0.22	0.998	4.88	0.4606	0.967
	4.0	8.7	17.9	0.999	8.44	0.0102	0.997
AlOOH–2%Fe_3_O_4_	3.0	20.3	0.18	0.999	4.30	0.4665	0.985
	4.0	7.8	1.00	0.986	6.48	0.0513	0.951

The maximum sorption capacity of 21.0 mg/g is achieved for pure AlOOH at pH 3.0. The sorption capacity decreases slightly (20.3 mg/g) for magnetic AlOOH–2%Fe_3_O_4_ powder at pH 3.0. It is noted that adsorbents with a magnetite content above 10 wt.% are difficult to separate from purified water. This is due to the increased dispersion of the composites (as evidenced by a significant increase in the specific surface area from 217 to 292 m^2^/g).

As follows, the increased sorption capacity of the powders is achieved by reducing both the magnetite content in the composites and the pH value. The results are because the degree of protonation of the surface of the oxides increases with decreasing pH. This allows chromium-containing anionic forms to be more efficiently adsorbed on a positively charged surface:

(8) S-OH + H^+^ ↔ S-OH_2_^+^,

(9) S-OH_2_^+^ + HCrO_4_^-^ ↔ SHCrO_4_ + H_2_O.

On the other hand, the addition of citrate-modified magnetite nanoparticles leads to a decrease in adsorption capacity due to the negative surface charge of these nanoparticles in the studied pH range (Figure 4). This leads to electrostatic repulsion of the chromium-containing anionic forms from the surface. In this regard, the adsorption capacity of the powders decreases with increasing magnetite content.

The experimental data were processed using theoretical Langmuir and Freundlich models (Figure 13a, Table 4). The Langmuir theory has the following fundamental assumptions: (1) it is monomolecular adsorption; (2) there are no interactions between adsorbate molecules; (3) adsorbent surface is homogenous. The Langmuir equation can be described by the linearized form [43]:

(10) $$\frac{C_{e}}{q_{e}}=\frac{1}{K_{L}q_{m}}+\frac{1}{q_{m}}C_{e},$$

*C*_*e*_ is the equilibrium Cr(VI) concentration (mg/L), *q*_*e*_ is the equilibrium Cr(VI) adsorption capacity (mg/g), *q*_*m*_ is the monolayer capacity (maximum adsorption capacity corresponding to the complete filling of the monolayer) (mg/g), *K*_*L*_ is the Langmuir constant (adsorption coefficient, which depends on the adsorption energy and temperature) (L/mg). *K*_*L*_ can be used to predict the affinity between the metal ions and the adsorbent.

Freundlich isotherm theory assumes the adsorbent surface being non-homogenous. The Freundlich equation can be described by the following formula [43]:

(11) $$lnq_{e}=lnK_{F}+\frac{1}{n}lnC_{e}.$$

here, *K*_*F*_ is Freundlich isotherm constant related to the adsorption capacity. The 1/*n* is considered as a standard to evaluate how favorable the adsorption process is. When 1/*n* ranges between 0 and 1, it demonstrates the adsorption process being favorable.

Based on the values of correlation coefficients (Table 4), it is obvious that the Langmuir model better describes all the obtained isotherms than the Freundlich model. This indicates the monolayer nature of the Cr(VI) adsorption on the surface of the obtained powders.

The possibility of cyclic use of adsorption-active material in water treatment is important for scalability and economic efficiency. For this purpose, adsorbed chromium compounds must be removed from the surface of the particles. This can be performed by increasing the pH (adding an alkali solution). After equilibrium is established, the adsorbent is washed and it can be reused for adsorption.

In this work, we examined the possibility of cyclic use of a magnetically separable AlOOH–2%Fe_3_O_4_ adsorbent (Figure 1b), which showed the highest adsorption capacity among composite powders (Figure 13b). It was shown that the adsorption properties are preserved, but a decrease in chromium adsorption is observed after each cycle (sorption decreases by 16% after the first cycle, and by 30% after the third cycle from the initial concentration).

A comparison of the adsorption characteristics of the obtained products with some similar systems (in composition, Table 5) was carried out. In general, the obtained products showed a sorption capacity comparable to or higher than analogs at the same pH values. Some research sources used a medium with pH of 2.0 [7]. However, low pH values can lead to the dissolution of both boehmite and magnetite. Thus, the authors showed that notable leaching was observed in acidic media (pH 2.0), reaching 12 wt. % and 0.5 wt.% for iron and aluminum respectively for the 3:1 ferria-alumina composite. The adsorption stability of products during cyclic use is comparable with analogs. For example, in Ref. [44] the sorption capacity is reduced by 35% after three cycles.

**Table 5:** Comparison of the sorption capacity of adsorbents.

Adsorbent	pH	Adsorption capacity *q*_m_, mg/g	Reference
AlOOH	3.0	21.0	This study
AlOOH–2%Fe_3_O_4_	3.0	20.3	This study
Hierarchical SiO_2_@γ-AlOOH spheres	3.0	4.5	[45]
Spherelike γ-Al_2_O_3_ microparticles	3.0	5.70	[46]
carbon/AlOOH	3.0	12	[47]
Sol-gel AlOOH	2.0	62.4	[7]
Sol-gel AlOOH–25%Fe_3_O_4_	2.0	52.9	[7]

Low cost, simplicity of preparation and using of the adsorbent are also important characteristics when scaling up adsorbent production. The studied AlOOH–Fe_3_O_4_ composites have a low cost due to the prevalence of elements and the availability of precursors, the non-use of difficult to decompose high molecular weight surfactants, and the absence of high-temperature production stages. Besides, all magnetite-containing powders (including AlOOH–2%Fe_3_O_4_) are magnetic, so that they can be easily separated from the purified liquid using a permanent magnet.

## 4 Conclusion

AlOOH–Fe_3_O_4_ nanocomposites were obtained using oppositely charged boehmite hydrosols and citric acid modified magnetite. Sols are oppositely charged in a wide range of pH 2.3–9.7, which contributes to their heteroaggregation. A study of heteroaggregation showed that as the concentration of Fe_3_O_4_ increases, the zeta potential decreased from +43 mV to -27 mV, passing through zero at a magnetite concentration of ~55 wt.%. High stability of the zeta potential and hydrodynamic diameter values up to 30 wt.% Fe_3_O_4_ was noted. Dispersed systems with a mass fraction of magnetite of up to 30% and more than 80% are sedimentation-stable, while sedimentation is observed in the range from 40 to 70 wt.% Fe_3_O_4_

AlOOH–Fe_3_O_4_ composite powders provide a high surface area (129–292 m^2^/g) with a maximum for AlOOH–10%Fe_3_O_4_ sample. The addition of a small number of magnetite nanoparticles leads to the formation of stable structures such as Fe_3_O_4_@AlOOH. Magnetite nanoparticles attached to the boehmite surface do not allow AlOOH particles to adhere strongly to each other during drying. As a result, powders with a highly porous structure and increased surface area are formed.

According to potentiometric titration, a relatively large boehmite particle is the core of composite particles for all the considered ratios of components. The adhesion of smaller spherical magnetite nanoparticles occurs on the surface of the boehmite particle due to acid-base (donor-acceptor) interactions.

The increased Cr(VI) adsorption capacity (up to 21 mg/g) on the powders is achieved by reducing both the magnetite content in the composites and the pH value. The presence of 2 wt.% magnetite in the nanocomposite leads to a slight (3.3%) decrease in adsorption capacity in comparison with pure boehmite at pH 3, but it imparts magnetic properties to this system.

Thus, low cost, simplicity of preparation, high adsorption capacity, and stability in cyclic use are the advantages of the resulting nanocomposites in comparison with similar systems. They can easily be separated from the purified liquid using a permanent magnet due to their magnetic properties.

## Declarations

### Author contribution statement

Vasily I. Mikhaylov: Conceived and designed the experiments; Performed the experiments; Analyzed and interpreted the data; Contributed reagents, materials, analysis tools or data; Wrote the paper.

Ilia S. Martakov, Petr A. Sitnikov: Performed the experiments; Analyzed and interpreted the data; Wrote the paper.

Evgeny Yu. Gerasimov: Performed the experiments; Contributed reagents, materials, analysis tools or data.

### Funding statement

This work was supported by the Council on grants of the President of the Russian Federation (MK-233.2019.3). This research was also partially supported by the 10.13039/501100006769Russian Science Foundation (19-73-10091, the study of morphology (TEM) and surface properties of magnetite and boehmite nanoparticles). Investigations were partially carried out using the equipment of the "Khimiya" Common Use Center (Institute of Chemistry, FRC Komi SC UB RAS).

### Data availability statement

Data included in article/supplementary material/referenced in article.

### Declaration of interests statement

The authors declare no conflict of interest.

### Additional information

No additional information is available for this paper.
